# Circulating proteins as predictors of cardiovascular mortality in end-stage renal disease

**DOI:** 10.1007/s40620-018-0556-5

**Published:** 2018-11-29

**Authors:** Tobias Feldreich, Christoph Nowak, Tove Fall, Axel C. Carlsson, Juan-Jesus Carrero, Jonas Ripsweden, Abdul Rashid Qureshi, Olof Heimbürger, Peter Barany, Peter Stenvinkel, Nicolas Vuilleumier, Philip A. Kalra, Darren Green, Johan Ärnlöv

**Affiliations:** 1 School of Health and Social Studies, Dalarna University, Falun, Sweden; 2 Division of Family Medicine, Department of Neurobiology, Care Sciences and Society, Karolinska Institutet, Huddinge, Sweden; 3 Department of Medical Sciences and Science for Life Laboratory, Uppsala University, Uppsala, Sweden; 4 Department of Medical Epidemiology and Biostatistics (MEB), Karolinska Institutet, Solna, Sweden; 5 Division of Medical Imaging and Technology, Department of Clinical Science, Intervention and Technology, Karolinska Institutet, Campus Flemingsberg, Stockholm, Sweden; 6 Division of Renal Medicine, Department of Clinical Science, Intervention and Technology (CLINTEC), Karolinska University Hospital, Stockholm, Sweden; 7 Department of Genetics, Laboratory Medicine and Pathology, Geneva University Hospitals, Geneva, Switzerland; 8 Department of Medical Specialties, Geneva Faculty of Medicine, Geneva, Switzerland; 9 Divison of Cardiovascular Sciences, The University of Manchester, Manchester Academic Health Sciences Centre, Manchester, UK; 10 Department of Renal, Medicine, Salford Royal NHS Foundation Trust, Stott Lane, Salford, UK

**Keywords:** CVD, ESRD, Proteomics

## Abstract

**Introduction:**

Proteomic profiling of end-stage renal disease (ESRD) patients could lead to improved risk prediction and novel insights into cardiovascular disease mechanisms. Plasma levels of 92 cardiovascular disease-associated proteins were assessed by proximity extension assay (Proseek Multiplex CVD-1, Olink Bioscience, Uppsala, Sweden) in a discovery cohort of dialysis patients, the Mapping of Inflammatory Markers in Chronic Kidney disease cohort [MIMICK; n = 183, 55% women, mean age 63 years, 46 cardiovascular deaths during follow-up (mean 43 months)]. Significant results were replicated in the incident and prevalent hemodialysis arm of the Salford Kidney Study [SKS dialysis study, n = 186, 73% women, mean age 62 years, 45 cardiovascular deaths during follow-up (mean 12 months)], and in the CKD5-LD-RTxcohort with assessments of coronary artery calcium (CAC)-score by cardiac computed tomography (n = 89, 37% women, mean age 46 years).

**Results:**

In age and sex-adjusted Cox regression in MIMICK, 11 plasma proteins were nominally associated with cardiovascular mortality (in order of significance: Kidney injury molecule-1 (KIM-1), Matrix metalloproteinase-7, Tumour necrosis factor receptor 2, Interleukin-6, Matrix metalloproteinase-1, Brain-natriuretic peptide, ST2 protein, Hepatocyte growth factor, TNF-related apoptosis inducing ligand receptor-2, Spondin-1, and Fibroblast growth factor 25). Only plasma KIM-1 was associated with cardiovascular mortality after correction for multiple testing, but also after adjustment for dialysis vintage, cardiovascular risk factors and inflammation (hazard ratio) per standard deviation (SD) increase 1.84, 95% CI 1.26–2.69, p = 0.002. Addition of KIM-1, or nine of the most informative proteins to an established risk-score (modified AROii CVM-score) improved discrimination of cardiovascular mortality risk from C = 0.777 to C = 0.799 and C = 0.823, respectively. In the SKS dialysis study, KIM-1 predicted cardiovascular mortality in age and sex adjusted models (hazard ratio per SD increase 1.45, 95% CI 1.03–2.05, p = 0.034) and higher KIM-1 was associated with higher CACscores in the CKD5-LD-RTx-cohort.

**Conclusions:**

Our proteomics approach identified plasma KIM-1 as a risk marker for cardiovascular mortality and coronary artery calcification in three independent ESRD-cohorts. The improved risk prediction for cardiovascular mortality by plasma proteomics merit further studies.

**Electronic supplementary material:**

The online version of this article (10.1007/s40620-018-0556-5) contains supplementary material, which is available to authorized users.

## Introduction

Chronic kidney disease (CKD) is a major public health problem worldwide [1] determining a significant burden of mortality, cardiovascular disease (CVD) being the leading cause of death [2–4]. Irrespective of therapeutic advances and improved care, end-stage renal disease (ESRD) patients have an up to 20-fold increased cardiovascular mortality risk compared to the general population [5]. Many of the traditional cardiovascular risk factors such as age, sex, dyslipidemia, diabetes mellitus and smoking do not appear to adequately explain the high cardiovascular risk in ESRD patients. As a consequence, managing ESRD-related CVD with standard clinical interventions is deemed suboptimal [6, 7]. Instead, non-traditional risk factors (such as mineral metabolism abnormalities, uremic toxins, and inflammation) contribute to cardiovascular pathology in ESRD [7–10], but little is known about which factors in the vascular milieu of hemodialysis patients are most important.

Recent years have witnessed unprecedented developments in the field of proteomics and process-specific biomarker panels for renal diseases [11–16]—techniques that could offer vital diagnostic and prognostic information as well as novel insights into mechanisms leading to CVD.

Our objective was to investigate the association between 92 cardiovascular proteins measured in plasma by a novel proteomics assay and the risk of cardiovascular mortality in prevalent hemodialysis patients, and to replicate the findings in an independent hemodialysis cohort. Furthermore we also wanted to assess whether plasma proteomics could improve the prediction of cardiovascular mortality beyond established risk factors. In order to provide additional mechanistic insights, a secondary aim was to use an independent cohort of CKD-stage 5 patients undergoing living donor renal transplantation (LD-RTx) with detailed data on cardiovascular phenotypes.

## Methods

### Discovery cohort, MIMICK

For the primary discovery analysis, we used the Mapping of Inflammatory Markers in Chronic Kidney disease study (MIMICK), a longitudinal study cohort consisting of 228 hemodialysis patients from six dialysis units in the Stockholm/Uppsala (Sweden) region. All subjects included had received dialysis treatment for ≥ 3 months, with a median follow-up period of 31 months (interquartile range, IQR 21–38). Survival, censored at transplantation, was determined from the day of examination. The patients were recruited from October 2003 through March 2004 and data on demographics, comorbidities and antihypertensive treatment were obtained by questionnaire or from hospital records. Venous blood samples were collected before the dialysis period, spun down immediately, and stored as EDTA plasma at -70 °C. High-sensitivity C-reactive protein (hsCRP) was measured by nephelometry. An immunometric assay on an Immulite Analyzer (Siemens Medical Solutions Diagnostics, Los Angeles, CA, USA) was used to quantify interleukin (IL)-6 in serum. Pentraxin 3 (PTX3) was determined by an ELISA kit (Perseus Proteomics, Tokyo, Japan). Routine biochemistry was performed in all of the six dialysis laboratory departments in the Stockholm/Uppsala region. In the current analysis, sufficient plasma samples for proteomics analysis were available for 183 of the patients. A detailed description of the study cohort has been previously reported [17, 18].

### Replication cohort, SKS dialysis study

As replication, we used the incident and prevalent hemodialysis arm of the Salford Kidney Study (SKS dialysis study), consisting of hemodialysis patients under the care of Salford Royal Hospital NHS Foundation Trust, United Kingdom. All patients received standard-hours, thrice weekly maintenance hemodialysis at Salford Royal Hospital or one of its satellite centers. The patients were enrolled between March 2012 and March 2014 with their written informed consent. Local ethical approval was granted (UK REC 05/Q1404/187), and the study complied with the Declaration of Helsinki.

The baseline clinical phenotype including demographic data, comorbidities, medications, and dialysis records was obtained from electronic patient medical records and patient self-reported questionnaires.

Blood samples were drawn from the dialysis circuit immediately before commencement of a dialysis session. Standard clinical tests were performed immediately and additional samples centrifuged and plasma and serum stored at − 80 °C. Such latter samples were used for KIM-1 analyses which were measured on citrated plasma by electrochemiluminescence, using the MESO QuickPlex SQ 120 automate from Mesoscale Discovery Systems (Rockville, MD, USA). A more detailed description of the cohort has been reported elsewhere [19].

### Secondary analyses, CKD5 patients undergoing living donor renal transplantation (LD-RTx)

For further pathophysiologic insight, we used a cross-sectional study consisting of 89 adult CKD5-LD-RTx at the Department of Transplantation Surgery at Karolinska University Hospital, Huddinge, Sweden. A comprehensive description of the study is available elsewhere [20]. Briefly, the median age was 46 years (range 24–62) and 37% were women. Pharmacological treatment, and previously diagnosed CVD was recorded. Out of the 89 participants, 39% were in pre-dialysis phase and 61% underwent either hemo- or peritoneal dialysis before RTx. Cardiac computed tomography (CT) scans were performed using a 64-channel detector scanner (LightSpeed VCT; General Electric Healthcare, Milwaukee, WI, USA) in cine mode. Calcium deposits in the coronary arteries (portraying both intima and media) were identified by an experienced radiologist [20]. An Advantage Workstation 4.4 (GE Healthcare) was used to process and analyze data, and Smartscore 4.0 (GE Healthcare) software was used to assess coronary artery calcium (CAC) scores. Values crossing the standard threshold of 130 Hounsfield units were considered indicative of calcified plaques. CAC scores were expressed in Agatston units (AU), and total CAC score was calculated as the sum of the CAC scores in the left main artery, left circumflex artery, right coronary artery, and the left anterior descending artery.

Informed consent was obtained from all patients involved, and the Regional Ethics Committee of the Karolinska Institute at the Karolinska University Hospital approved both study protocols.

### Proteomics

The Olink Proseek^®^ Multiplex Cardiovascular I^96X96^ kit (http://www.olink.com/) is a proximity extension assay (PEA) that measures the relative abundance of 92 cardiovascular proteins. For each protein, oligonucleotide-labeled antibody pairs bind to their specific epitopes on the protein surface [21, 22]. The complementary oligonucleotide sequences then give rise to DNA reporter sequences each barcoding their respective antigens. Using a Fluidigm Biomark™ HD real-time polymerase chain reaction (PCR) platform, we then quantified these amplicons. Mean intra- and inter-assay coefficients of variation are 8 and 12%, respectively, with a reported inter-site variation of 15% [22]. Log_2_-scaled normalized protein expression values were adjusted by a negative control sample. Higher expression values correspond to higher protein levels, but are not an absolute quantification of protein concentrations.

### Outcome definition

In the MIMICK cohort, the patients were followed from the inclusion date until renal transplantation or death or completion of 60 months of follow-up. Causes of death were established by the death certificate issued by the attending physician. Cardiovascular mortality was defined according to International Classification of Diseases (10th revision) codes I00–I99. Follow-up in the SKS dialysis study was from the date of a study protocol echocardiogram (again between March 2012 and March 2014) until death, transplantation, re-location, or August 10th 2016. Causes of death and events were independently verified by two blinded assessors.

### Statistical analysis

Analyses were carried out using STATA 12 (StataCorp, College Station, TX, USA) and R v.3.3.2.

#### Primary analyses

We used MIMCK-1 to investigate associations between the 92 proteins and cardiovascular mortality in an age and sex-adjusted Cox proportional hazard regression (Model A). A p value < 0.00054 (Bonferroni correction 0.05/92 proteins) was considered statistically significant. Protein values were transformed to a mean of 0 and standard deviation of 1. We then replicated the significant associations in an independent cohort, SKS dialysis study, of hemodialysis patients using age and sex-adjusted Cox proportional hazard regression.

#### Secondary analyses

For proteins that were significantly associated with cardiovascular mortality in the primary analysis, we performed additional multivariable Cox regression analyses in MIMICK adjusting for the following variables:

B. Age, sex, and dialysis vintage to determine if the associations were independent of general characteristics and time on dialysis.
C. Age, sex, dialysis vintage, CVD, and N-terminal prohormone of brain natriuretic peptide (NT-proBNP) to determine if the associations were independent of prevalent CVD and heart dysfunction.
D. Age, sex, dialysis vintage, CVD, NT-proBNP, and cardiovascular risk factors—diabetes mellitus (DM), body mass index (BMI), high density lipoproteins (HDL), low density lipoproteins (LDL), and smoking—to determine if the associations were independent of established cardiovascular risk factors measured in clinical practice.
E. Age, sex, dialysis vintage, CVD, NT-proBNP, cardiovascular risk factors (DM, BMI, HDL, LDL, and smoking), and inflammatory markers (hsCRP, IL-6, and PTX3) to determine if the associations were independent of all factors above and significant markers of inflammation.

In these analyses, a p value < 0.05 was considered statistically significant.

In the CKD5-LD-RTx cohort, we also performed cross-sectional analyses between the significant proteins from the discovery replication analyses and coronary artery calcification by calculating the Spearman correlation coefficient and applying linear regression adjusted for age and sex. In these analyses, coronary artery calcification was included as a categorical variable (CAC < 400, CAC 400–1000 and CAC > 1000 Hounsfield units).

#### Risk prediction

To assess whether adding the proteomics data to an established risk score can improve the prediction of cardiovascular mortality, we used Lasso penalized Cox proportional hazards regression [23] to select a parsimonious model that maximized discrimination performance whilst minimizing the number of proteins used for prediction. We used a modified version of the AROii CVM-score [24] (http://aro-score.askimed.com/) as our base model. The variables available in our dataset that were also included in the ARO risk score were: age, sex, history of CVD, DM, BMI, CRP, smoking status, hemoglobin, ferritin, serum albumin, serum calcium, serum creatinine, history of malignancy and cause of renal disease (diabetes, glomerulonephritis or other). The remaining variables in the ARO risk score (dialysis-related variables) had not been retrieved in the majority of participants and could not be included. However, even though the AROii CVM-score performs best when all components of the score are included, its use is encouraged even in cases where some variables are missing [24]. We forced all available ARO risk score variables into the model and implemented Lasso selection with 10-fold cross-validation and default parameters with the cv.glmnet function in the R package glmnet. The sample was randomly split into a 60% training set and 40% validation set. The Lasso model was trained in the training set and all proteins there were included in the iteration that converged on the smallest cross-validated error were selected and tested in the separate 40% validation sample. Harrell’s C-index in the validation sample was calculated with the survConcordance function and stored. We repeated this procedure in 1000 random iterations and retained the top 50% of models ranked by C-index. The number of times each protein was included in the predictor selection was plotted in histograms to identify cut-off frequencies between top predictors and less important predictors; the more often a protein was selected by one of these top-performing models, the higher was its presumed importance for predicting the outcome. Finally, we implemented a Cox regression model in the total sample with the final set of top predictors added to the risk score variables to assess prediction performance (C-index) and goodness-of-fit (log-likelihood test). The prediction analyses were performed in the MIMICK cohort only.

## Results

### Baseline characteristics

A summary of general characteristics of the MIMICK, SKS dialysis study and CKD5-LD-RTx cohorts is presented in Table 1.

**Table 1:** Baseline characteristics of patients from the different cohorts

Variable	MIMICK-cohort	SKS dialysis study	ESRD-RT-cohort
N	183	186	89
Sex (% female)	55	73	37
Age (years)	63 ± 14	62 ± 14	46 ± 14
BMI (kg/m^2^)	25 ± 5.0	28 ± 6.2	25 ± 2.0
Dialysis vintage, months	44 ± 49	31 ± 3.5	1.1 ± 1.8
hsCRP (mg/l)	6.4 (2.6–22)	18 ± 39	0.8 (0.4–2.4)
IL-6 (pg/ml)	8.9 (5.0–15)	N.A.	1.1 (0.5–2.1)
PTX3 (ng/ml)	10 (7.1–17)	N.A.	3.9 (2.0–6.4)
HDL (mmol/l)	1.4 ± 0.5	N.A.	1.4 ± 0.5
LDL (mmol/l)	2.6 ± 0.9	2.0 ± 1.1	2.5 ± 0.95
Hemoglobin (g/l)	118 ± 13	N.A.	115 ± 14
Ferritin (µg/l)	485 ± 361	550 ± 354	N.A.
Serum albumin (g/l)	35 ± 5	39 ± 3.8	36 ± 3.6
Serum calcium (mmol/l)	2.5 ± 0.2	2.4 ± 0.17	2.3 ± 0.2
Creatinine (µmol/l)	770 ± 211	716 ± 254	759 ± 237
NT-pro-BNP (pg/l)	14 ± 13	0.4 ± 0.5	5.2 ± 0.4
Smoking (%)	17	N.A.	46
Diabetes mellitus (%)	25	71	14
Cardiovascular disease (%)	19	42	18

Normally distributed continuous variables are presented as mean ± standard deviation, skewed continuous variables as median (interquartile range) (25th–75th percentile), and categorical variables as percentage

*MIMICK* Mapping of Inflammatory Markers in Chronic Kidney disease, *SKS dialysis study* incident and prevalent hemodialysis arm of the Salford Kidney Study, *ESRD* end-stage renal disease, *hsCRP* high-sensitivity C-reactive protein, *IL-6* interleukin 6, *PTX3* pentraxin 3, *NSAID* nonsteroidal anti-inflammatory drugs, *ACE* angiotensin-converting enzyme, *ASA* acetylsalicylic acid, *ACEi/ARB* angiotensin-converting enzyme inhibitor/angiotensin receptor blocker, *HDL* high density lipoproteins, *LDL* low density lipoproteins, *N.A*. not available

After adjusting for age and sex in the MIMICK cohort, 11 proteins showed nominally significant associations with cardiovascular mortality. In the order of level of significance, these included KIM-1, matrix metalloproteinase (MMP)-7, tumor necrosis factor receptor 2 (TNFR2), IL-6, MMP-1, brain-natriuretic peptide (BNP), suppression of tumorigenicity 2 (ST2), hepatocyte growth factor (HGF), TNF-related apoptosis inducing ligand receptor-2 (TRAIL-R2), spondin-1, and fibroblast growth factor 25 (FGF25) (Table 2). The association between all 92 proteins and cardiovascular mortality is depicted in supplementary figure 1.

After Bonferroni correction for multiple testing, only plasma kidney injury molecule-1 (KIM-1) was significantly associated with cardiovascular mortality (hazard ratio, HR, per SD increase, 1.80, 95% confidence interval (CI) 1.33–2.44, p < 0.0001. In the SKS replication cohort, KIM-1 was also significantly associated with an increased risk of cardiovascular mortality (HR per SD increase 1.45, 95% CI 1.03–2.05, p = 0.034). In additional multivariable models in the MIMICK cohort, raised KIM-1 levels were significantly associated with cardiovascular mortality after adjustment for age, sex, dialysis vintage, CVD, NT-proBNP, cardiovascular risk factors (DM, BMI, HDL, LDL, and smoking), and inflammatory markers (hsCRP, IL-6, and PTX3; model A–E, Table 3).

**Table 2:** Associations between circulating protein markers and cardiovascular mortality in hemodialysis patients (MIMICK cohort)

Cardiovascular mortality	Age and sex adjusted	
Protein	HR (95% CI)	p
Kidney injury molecule-1	1.80 (1.33–2.44)	0.0001
Matrix metalloproteinase-7	2.54 (1.43–4.52)	0.002
Tumour necrosis factor receptor 2	12.6 (2.19–66.0)	0.004
Interleukin 6	1.56 (1.14–2.15)	0.005
Matrix metalloproteinase-1	1.62 (1.13–2.32)	0.008
Brain natriuretic peptide	1.62 (1.03–2.33)	0.009
Suppression of tumorigenicity 2	1.63 (1.13–2.35)	0.009
Hepatocyte growth factor	1.37 (1.05–1.79)	0.02
TNF-related apoptosis-inducing ligand receptor 2	1.87 (1.10–3.18)	0.02
Spondin-1	1.43 (1.05–1.94)	0.02
Fibroblast growth factor 25	3.09 (1.03–9.22)	0.04

HR and 95% CI are given for an age and sex adjusted model

p < 0.05 was considered statistically significant

*CI* confidence interval, *HR* hazard ratio, *MIMICK* Mapping of Inflammatory Markers in Chronic Kidney disease cohort, *TNF* tumor necrosis factor

**Table 3:** Associations between circulating protein marker kidney injury molecule-1 (KIM-1) and cardiovascular mortality in hemodialysis patients (MIMICK cohort)

Protein	Cardiovascular mortality	
KIM-1	HR (95% CI)	p
Model A^a^	1.80 (1.33–2.44)	0.0001
Model B^b^	1.75 (1.27–2.42)	0.0006
Model C^c^	2.12 (1.50–3.01)	0.00002
Model D^d^	2.07 (1.42–3.02)	0.0001
Model E^e^	2.12 (1.43–3.16)	0.0002

Values are hazard ratios (HR) with 95% confidence intervals (CI) with hemodialysis as dependent variable and the 92 protein markers as independent variables in separate models

p < 0.05 was considered statistically significant

*MIMICK* Mapping of Inflammatory Markers in Chronic Kidney disease cohort, *CVD* cardiovascular disease, *NT-proBNP* N-terminal prohormone of brain natriuretic peptide, *DM* diabetes mellitus, *BMI* body mass index, *HDL* high density lipoproteins, *LDL* low density lipoproteins, *CRP* C-reactive protein, *IL-6* interleukin 6, *PTX3* pentraxin-related protein

^a^Adjusted for age and sex

^b^Adjusted for age, sex, dialysis vintage

^c^Adjusted for age, sex, dialysis vintage, CVD, and NT-proBNP

^d^Adjusted for age, sex, dialysis vintage, CVD, NT-proBNP, and cardiovascular risk factors (DM, BMI, HDL, LDL, and smoking)

^e^Age, sex, dialysis vintage, CVD, NT-proBNP, cardiovascular risk factors (DM, BMI, HDL, LDL, and smoking), CRP, IL-6, and PTX3

In the mechanistic analyses in the CKD5-LD-RTx-cohort, there was a significant correlation between higher plasma KIM-1 and higher CAC-score (Spearman rho = 0.27, p = 0.008). A significant association was also seen between higher plasma levels of KIM-1 and higher CAC-score when adjusting for age and sex in linear regression (β-coefficient per SD increase in protein abundance 0.11, 95% CI 0.01–0.20, *p* = 0.03).

**Fig. 1:**
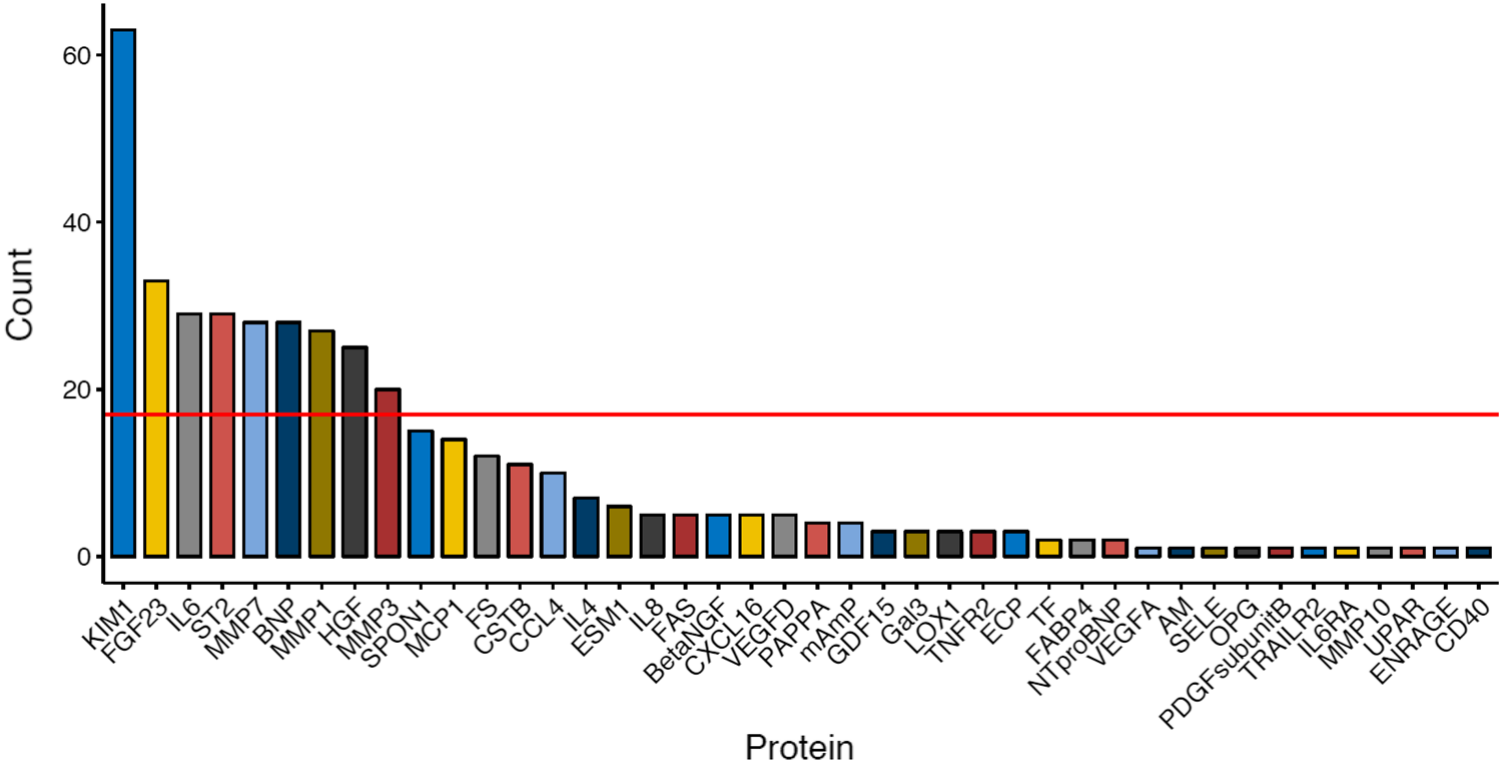
Histogram showing proteins most frequently selected as top predictors by the 500 best-performing Lasso penalized Cox models, e.g. protein KIM-1 was selected by 63 of the top models. The red line indicates the arbitrary cut-off for the prediction models chosen in this study for KIM-1, as well as the next most frequent proteins, FGF-23, IL-6, ST-2, MMP-7, BNP, MMP-1, HGF and MMP-3. Proteins that are not shown in the histogram were not selected by any of the 500 best-performing solutions

In the MIMICK cohort, we implemented Lasso penalized regression across 1000 iterations each splitting the total sample into a 60% training set used to build the Lasso model, and a separate 40% validation set used to estimate the C-index. A clear cut-off that selected KIM-1 as the most important protein was apparent in a histogram of how often proteins had been selected by the best-performing 500 models (Fig. 1). Protein KIM-1 was selected by 63 of the top models. A second cut-off for top predictors was apparent (marked in Fig. 1), that selected KIM-1, FGF-23, IL-6, ST-2, MMP-7, BNP, MMP-1, HGF and MMP-3.

In the total sample, the baseline model (AROii CVM-score) achieved a C-index of 0.777 (95% CI 0.692–0.862). The addition of KIM-1 improved prediction performance to C = 0.799 (95% CI, 0.714–0.884) and led to better model fit (p = 0.0012). Addition of the nine proteins that were nominally associated with CVD mortality to the AROii CVM-score achieved C = 0.823 (95% CI, 0.738–0.909) and a better model fit (p = 4.56 × 10^−4^).

## Discussion

We used a novel targeted proteomics assay to explore associations between 92 cardiovascular disease-related proteins in plasma and cardiovascular mortality in a discovery cohort of prevalent hemodialysis patients. Eleven proteins were associated with cardiovascular death at nominal significance. Only plasma KIM-1—also denoted as T cell immunoglobulin and mucin domain (TIM) or Hepatitis A virus cellular receptor 1 (HAVCR-1)—predicted cardiovascular mortality after correction for multiple testing. This association remained statistically significant even after adjustment for age, sex, dialysis vintage, prevalent CVD, NT-proBNP, other cardiovascular risk factors, and various inflammatory markers. We then replicated the significant findings in an independent cohort in which KIM-1 also showed a significant association with cardiovascular mortality after adjusting for sex and age. Furthermore, higher plasma KIM-1 was associated with increased coronary artery calcification in a cross-sectional analysis in an independent cohort of CKD 5/5D patients undergoing living donor renal transplantation. The addition of plasma KIM-1, alone, or of a 9-protein risk score to the modified AROii CVM-score appeared to improve the risk prediction for cardiovascular mortality, but larger studies are needed to draw firm conclusions on the clinical utility.

Previous large-scale proteomic efforts in CKD patients are scarce and have primarily utilized urine samples for the proteomics analyses [25–27], with a few exceptions [28]. To a limited degree, small proteomics-based studies have been performed using plasma samples in CKD5 patients [29].

KIM-1, a type I cell membrane glycoprotein initially identified in the African green monkey, has been shown to regulate immune cell responses to infections [30], autoimmune and allergic diseases [31] and antitumor effects [32]. The expression of KIM-1 is highly upregulated in the proximal tubule of the kidney after injury, and urinary levels of KIM-1 have been demonstrated as a promising biomarker in both acute and chronic kidney disease as well as a predictor for cardiovascular outcomes in CKD patients [33–37] and in the general population [38]. However, few studies have evaluated blood-borne KIM-1 as a biomarker. Two previous cross-sectional reports demonstrated elevated plasma KIM-1 levels in both acute and chronic kidney disease patients [39] and higher levels with increasing severity of CKD [40]. In longitudinal analyses, higher plasma KIM-1 was associated with a more rapid decline in glomerular filtration rate (GFR) [40] and a greater risk for ESRD [41]. Importantly, we are not aware of any previous study reporting the association between plasma KIM-1 and cardiovascular mortality in hemodialysis patients.

The detection of KIM-1 in plasma or urine has been attributed to loss of tubular cell polarity, compromised transepithelial permeability, and cytoskeletal disruption in renal microvascular cells [40]. Several other studies have pointed to an upregulated expression and increased release of KIM-1 in renal tubular cells after injury [36, 42, 43]. The potential expression of KIM-1 in other tissues, such as within the vasculature, needs consideration since all patients in this study had a narrow and very low range of eGFR. Our finding of an association between plasma KIM-1 and coronary artery calcification in the CKD5-LD-RTx-cohort implies that circulating KIM-1 is also a marker for atherosclerotic disease, which might explain the strong independent association with cardiovascular mortality [44]. KIM-1 has been implicated in the mitogen-activated protein kinase (MAPK) signaling pathway [39] which is involved in the activation of macrophages in kidney injury and fibrosis [45] but also in cardiovascular pathology with both promoting and suppressing effects [46–48]. Whether circulating KIM-1 reflects these pathways remains to be established.

Eleven of the 92 proteins showed nominally significant associations with cardiovascular mortality. Although we could not establish causality, possible underlying mechanisms might involve inflammation (IL-6, and ST2), extracellular matrix remodeling (MMP-1 and MMP-7), apoptosis (TRAIL-R2), increased ventricular overload due to hydric retention (NT-proBNP), and cell growth, cell motility, and morphogenetic (HGF) properties [49, 50].

Better discrimination of high risk vs. low risk hemodialysis patients could be of great value in tailoring individualized treatments, in decision-making for transplantation, but also to refine inclusion and exclusion criteria for clinical trials thus enabling more powerful cost-effective designs. For this purpose, a new risk score was recently introduced (the AROii CVM-score) [24]. Even though all components of the AROii CVM-score were not available in the MIMICK-cohort, the modified version of the score performed at least as well in our study as the complete score did in the original article C-statistics of the modified AROii CVM-score in MIMICK were 0.78 compared to 0.72–0.74 for the complete score in the original article [24]. As a clear improvement in C-statistics was seen when adding data on plasma KIM-1 or the nine most informative plasma proteins to the modified AROii CVM-score, our data support the notion that proteomic profiling has potential for improving cardiovascular risk prediction in hemodialysis patients. Yet, these findings should be interpreted with caution as our study was underpowered to detect statistically significant improvements in C-statistics.

Strengths of our study include the longitudinal design and the fact that we were able to replicate the association between plasma KIM-1 and relevant cardiovascular phenotypes in independent patient populations. Limitations include the fact that the PEA technique does not allow absolute quantification of the proteins, and so determining cut-off values of the different proteins is less straightforward in a clinical setting. Second, the delay between sampling and analysis may have affected protein levels, but sample collection was undertaken in a consistent fashion and samples stored unthawed at a minimum of − 70 °C, which should keep pre-analytical biases to a minimum. If anything, any such bias would dilute associations. In fact, the associations were identical after adjustments for freezer time (data not shown). Finally, the generalizability of our results may be limited since our study sample predominantly consisted of individuals of particular age groups and European descent.

Our proteomics approach identified plasma KIM-1 as a promising prognostic marker that merits further investigation. Our results imply that KIM-1 is generated also in tissue(s) other than the kidney and that it may have a potential pathogenic role in premature vascular ageing processes. Furthermore, our data encourage additional efforts to evaluate the utility of targeted proteomic profiling in routine clinical care of hemodialysis patients.

## Electronic supplementary material

Below is the link to the electronic supplementary material.

Supplementary material 1 (DOCX 16 KB)
