# Supplementary material for: Circulating proteins as predictors of cardiovascular mortality in end-stage renal disease
Source: J Nephrol. 2018 Nov 29;32(1):111–9. doi: 10.1007/s40620-018-0556-5 (PMC6373380; doi:10.1007/s40620-018-0556-5)
Supplement: Supplementary file 1 — Supplementary material 1 (DOCX 16 KB) [file 40620_2018_556_MOESM1_ESM.docx]

**Supplementary table 1**. Cross sectional associations between the multiplex cardiovascular kit 92 proteins and cardiovascular mortality using linear regression models adjusted for age and sex in the MIMICK-cohort.

| **Protein** | **HR (95% CI)** | **p-value** |
| --- | --- | --- |
|  |  |  |
| TIM | 1.80 (1.33-2.44) | 0.0001407 |
| MMP7 | 2.54 (1.43-4.52) | 0.0015203 |
| TNFR2 | 12.02 (2.19-66) | 0.0042006 |
| IL6 | 1.56 (1.14-2.15) | 0.0054943 |
| MMP1 | 1.62 (1.13-2.32) | 0.0083206 |
| BNP | 1.62 (1.13-2.33) | 0.0085507 |
| ST2 | 1.63 (1.13-2.35) | 0.0094972 |
| HGF | 1.37 (1.05-1.79) | 0.0200888 |
| TRAILR2 | 1.87 (1.10-3.18) | 0.0217187 |
| SPON1 | 1.43 (1.05-1.94) | 0.0228027 |
| FGF23 | 3.09 (1.03-9.22) | 0.0437584 |
| MCP1 | 1.62 (0.97-2.71) | 0.063075 |
| VEGFD | 1.52 (0.96-2.40) | 0.0748637 |
| AM | 2.31 (0.91-5.90) | 0.079265 |
| KLK6 | 0.7 (0.47-1.05) | 0.0856939 |
| CCL3 | 0.72 (0.49-1.07) | 0.1030041 |
| GDF15 | 1.35 (0.94-1.95) | 0.108237 |
| IL8 | 1.31 (0.94-1.82) | 0.1121581 |
| TNFR1 | 2.28 (0.81-6.41) | 0.1169773 |
| CX3CL1 | 0.62 (0.34-1.14) | 0.1207369 |
| UPAR | 2.05 (0.82-5.17) | 0.1266709 |
| ENRAGE | 1.20 (0.95-1.53) | 0.1272105 |
| BetaNGF | 1.34 (0.91-2.00) | 0.1425565 |
| CSTB | 2.61 (0.71-9.56) | 0.1486276 |
| SRC | 1.39 (0.89-2.18) | 0.1520471 |
| CXCL16 | 1.31 (0.90-1.89) | 0.1552593 |
| CSF1 | 1.71 (0.81-3.63) | 0.1600384 |
| SELE | 1.34 (0.89-2.02) | 0.1604891 |
| TRANCE | 0.78 (0.55-1.11) | 0.1722394 |
| CASP8 | 0.66 (0.36-1.21) | 0.1824959 |
| FS | 1.19 (0.91-1.55) | 0.2050886 |
| FABP4 | 1.77 (0.72-4.36) | 0.2172612 |
| SCF | 0.80 (0.57-1.14) | 0.2180945 |
| Dkk1 | 0.80 (0.55-1.15) | 0.2309339 |
| MMP12 | 1.29 (0.85-1.96) | 0.2366508 |
| FAS | 1.25 (0.86-1.81) | 0.2373445 |
| mAmP | 1.17 (0.88-1.56) | 0.2894014 |
| TRAIL | 0.81 (0.56-1.19) | 0.2904816 |
| LOX1 | 1.18 (0.87-1.60) | 0.2941473 |
| IL27A | 1.22 (0.84-1.78) | 0.2996882 |
| PAPPA | 1.18 (0.86-1.62) | 0.3004926 |
| CXCL6 | 0.80 (0.51-1.26) | 0.3276706 |
| CCL20 | 1.16 (0.86-1.57) | 0.333046 |
| MB | 0.84 (0.60-1.19) | 0.3386096 |
| CHI3L1 | 0.85 (0.60-1.20) | 0.355306 |
| GAL | 0.84 (0.57-1.23) | 0.3572554 |
| ITGB1BP2 | 0.64 (0.22-1.83) | 0.4069177 |
| OPG | 1.20 (0.76-1.92) | 0.4335457 |
| VEGFA | 1.36 (0.63-2.98) | 0.4351293 |
| tPA | 1.18 (0.77-1.82) | 0.4373079 |
| TIE2 | 0.87 (0.58-1.29) | 0.4761232 |
| HSP27 | 1.16 (0.77-1.74) | 0.4778488 |
| NEMO | 0.83 (0.49-1.40) | 0.4806967 |
| REN | 0.89 (0.65-1.22) | 0.4813995 |
| IL6RA | 1.15 (0.78-1.69) | 0.4828532 |
| GH | 1.15 (0.76-1.76) | 0.5036464 |
| AGRP | 0.86 (0.56-1.33) | 0.5082098 |
| PTX3 | 1.15 (0.76-1.75) | 0.5167953 |
| MMP3 | 1.14 (0.76-1.73) | 0.5235526 |
| NTproBNP | 1.45 (0.46-4.50) | 0.5247955 |
| ESM1 | 1.12 (0.79-1.58) | 0.5337852 |
| MPO | 0.90 (0.64-1.27) | 0.5399903 |
| hK11 | 0.87 (0.54-1.38) | 0.5537565 |
| HBEGF | 0.81 (0.39-1.67) | 0.5617187 |
| RETN | 0.89 (0.58-1.35) | 0.5697793 |
| CCL4 | 1.07 (0.81-1.43) | 0.6339074 |
| RAGE | 1.10 (0.71-1.70) | 0.6795565 |
| PlGF | 0.87 (0.43-1.73) | 0.6852745 |
| ECP | 1.07 (0.77-1.48) | 0.685736 |
| IL16 | 0.92 (0.58-1.45) | 0.7138655 |
| LEP | 0.94 (0.69-1.29) | 0.7146233 |
| PECAM1 | 1.07 (0.74-1.54) | 0.7160675 |
| SIRT2 | 1.07 (0.75-1.52) | 0.7273142 |
| PDGFsubunitB | 1.06 (0.73-1.55) | 0.7500621 |
| CXCL1 | 1.05 (0.75-1.49) | 0.7670017 |
| CD40 | 1.13 (0.50-2.55) | 0.7680025 |
| CTSL1 | 1.06 (0.71-1.59) | 0.782456 |
| TF | 1.07 (0.63-1.84) | 0.7931644 |
| PSGL1 | 1.10 (0.51-2.38) | 0.8082271 |
| IL18 | 0.96 (0.67-1.39) | 0.8418693 |
| CD40L | 0.96 (0.62-1.48) | 0.8548617 |
| IL1ra | 0.98 (0.72-1.33) | 0.873789 |
| TNFSF14 | 1.02 (0.78-1.34) | 0.8834885 |
| CA125 | 0.97 (0.67-1.42) | 0.8865508 |
| EGF | 1.03 (0.71-1.49) | 0.8900577 |
| TM | 0.97 (0.58-1.62) | 0.906372 |
| MMP10 | 0.98 (0.68-1.41) | 0.9087857 |
| CTSD | 1.01 (0.72-1.44) | 0.9344096 |
| Gal3 | 0.99 (0.70-1.39) | 0.9373022 |
| IL4 | 0.99 (0.65-1.50) | 0.9595469 |
| PRL | 1.01 (0.74-1.37) | 0.9622709 |
| PAR1 | 1.02 (0.43-2.42) | 0.9637327 |
